# A laparoscopic resection of lung cancer metastatic to transverse colon: A case report and review of the literature

**DOI:** 10.1002/ccr3.2296

**Published:** 2019-07-21

**Authors:** Ryota Koyama, Nozomi Minagawa, Yoshiaki Maeda, Toshiki Shinohara, Tomonori Hamada

**Affiliations:** ^1^ Department of Gastrointestinal Surgery Hokkaido Cancer Center Sapporo Japan

**Keywords:** laparoscopic surgery, lung cancer, lung cancer metastatic to colon, palliative surgery

## Abstract

Metastatic colon cancer from primary lung cancer is usually a part of systemic dissemination, suggesting limited prognosis. However, surgical intervention for symptomatic patients such as hemorrhage is sometimes required. Surgeons must carefully determine the surgical indication in view of prognosis and quality of life.

## 1 BACKGROUND

Lung cancer often metastasizes to the lung itself, liver, brain, adrenal gland, and bone. However, the colon is a rare site for metastasis. Lung cancer metastatic to the colon is commonly the end‐stage presentation with systemic dissemination, and the case requiring surgical intervention is rare. Long‐term survival cases are reported after resection the colorectal metastasis with curative intent, but the prognosis is generally poor. The surgical indication of palliative surgery must be carefully considered when making a decision.

## 2 CASE PRESENTATION

The 68‐year‐old male received left upper pneumonectomy for lung cancer with lymph node dissection (ND2a‐1, pT2bN0M0, Stage2A) 1 year ago. The lung cancer consisted of moderately differentiated squamous cell carcinoma with keratinization and intercellular bridge. Immunohistochemistry showed positive for cytokeratin 7 (CK7), and negative for cytokeratin 20 (CK20) and caudal‐related homeobox 2 (CDX2).

**Figure 1:**
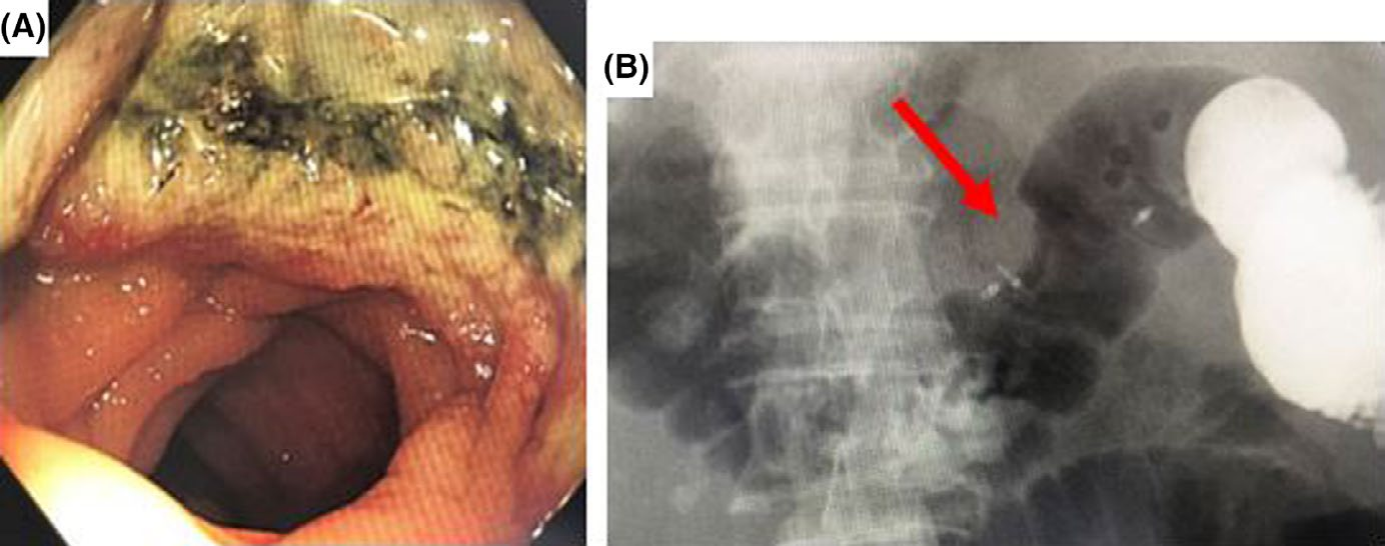
Colonoscopy and contrast enema finding. A, A hemorrhagic ulcerated lesion with marginal elevation was found at the transverse colon near the splenic flexure on colonoscopy. B, Arc‐shaped lateral deformity was found at the same site on contrast enema study (arrow)

Five months after the surgery for lung cancer, he developed dyspnea, and fecal occult blood test was positive. Colonoscopy revealed a hemorrhagic ulcerated lesion with marginal elevation in the transverse colon (Figure 1A). Chemotherapy for the recurrence of lung cancer was required; however, surgical intervention for progression of anemia due to the advanced tumor in the transverse colon was considered before initiating chemotherapy.

He had a medical history of old myocardial infarction (18 years prior; postpercutaneous cardiac intervention, and long‐term use of antithrombotics (including ticlopidine hydrochloride and dabigatran etexilate), diabetes mellitus, hypertension, and dyslipidemia. He had no family history regarding malignancy. On admission, his height was 182 cm, and weight was 81 kg. Blood pressure was 112/66, heart rate was 82 beats per minute, body temperature was 36.6°C, and oxygen saturation was 96% on room air, Glasgow Coma Scale was 15 points, and performance status was 2. The abdomen was soft and flat without any palpable mass.

Laboratory evaluation showed anemia (hemoglobin 9.7 g/dL), extremely low albumin (1.9 g/dL) and choline esterase (107 U/L), suggesting poor nutrition. Renal function was slightly lowered (blood urea nitrogen 14.1 mg/dL, creatinine 1.13 mg/dL), and there were high inflammatory markers (WBC 9300/mm^3^, C‐reactive protein 10.27 mg/dL). Electrolytes were normal, and tumor markers (squamous cell carcinoma antigen, neuron‐specific enolase, sialyl Lewis Xi antigen, progastrin releasing peptide, carcinoembryonic antigen, and carbohydrate antigen 19‐9) were within normal limits except for slight elevation of soluble cytokeratin 19 fragment of 12.1 ng/mL (range 0‐2.8 ng/mL). Electrocardiogram showed incomplete right bundle branch block and old myocardial infarction. Echocardiogram showed sustained systolic function of ejection fraction (56.9%) and asynergy with dyskinesis around the apex of the heart, akinesis and thinning of the anterior wall. The lateral deformity was observed by contrast enema using gastrografin, suggesting advanced tumor (Figure 1B).

Biopsy of the tumor in the transverse colon showed cancer cells in a small gland formation proliferating mainly in the submucosal layer. Immunohistochemistry was positive for CK7, and negative for CK20, CDX2, and thyroid transcription factor‐1 (TTF‐1), which was incompatible with primary colon cancer, leading to the preoperative diagnosis of lung cancer metastatic to the colon.

**Figure 2:**
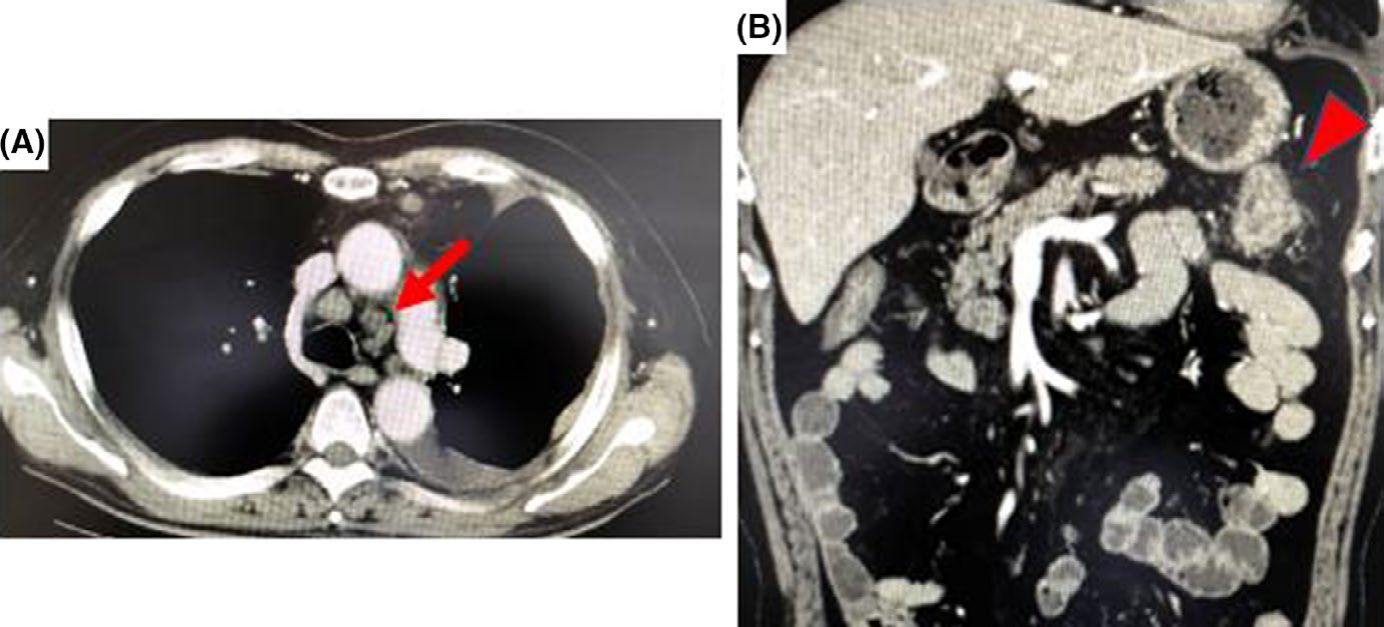
Contrast computed tomography finding. A, Swollen lymph nodes in the left hilum of lung (arrow) and left pleural effusion. B, Thickening of the wall of transverse colon (arrow head) indicating the tumor location and some nearby swollen lymph nodes

Chest CT showed multiple dilated lymph nodes in the left lung hilum and mediastinum (Figure 2A). Left pleural effusion and irregular thickening of the left pleura were also observed, suggesting left pleural dissemination. Abdominal CT showed thickened left side transverse colon with several nearby dilated lymph nodes (Figure 2B). No ascites and peritoneal dissemination were observed. Fluorodeoxyglucose positron emission tomography showed enhanced uptake in the mediastinum, left lung hilum, and the sites of thickened pleura. The tumor in the transverse colon showed limited uptake with SUVmax of 11.76. In summary, preoperative diagnosis of lung cancer metastatic to the colon was determined.

Differential diagnosis included primary colon cancer; however, past medical history, regional relapse in the left lung hilum and mediastinum, and left pleural effusion suggested lung cancer metastatic to colon.

On admission, patient was given 13.5 g piperacillin/tazobactam and 1500 mg metronidazole to decrease inflammation. A chest tube was inserted into the left pleural space to control left pleural effusion. Anemia was treated with transfusion. Antithrombotics were discontinued, and heparin replacement was initiated. After the systemic condition improved, laparoscopic resection was successfully performed. Operation time was 102 minutes, and blood loss was 5 mL.

**Figure 3:**
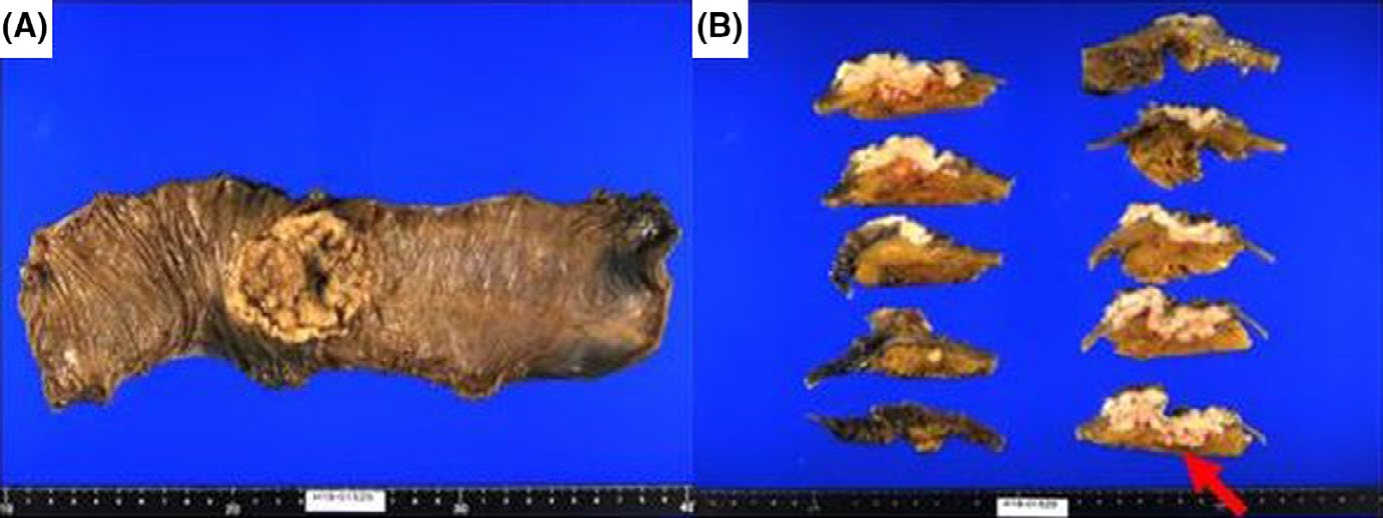
Resected specimen. A, An ulcerated lesion with marginal elevation of the transverse colon measuring 6.5 × 5.5 cm was resected. B, Cancer was exposed on surface of the serosa (arrow)

**Figure 4:**
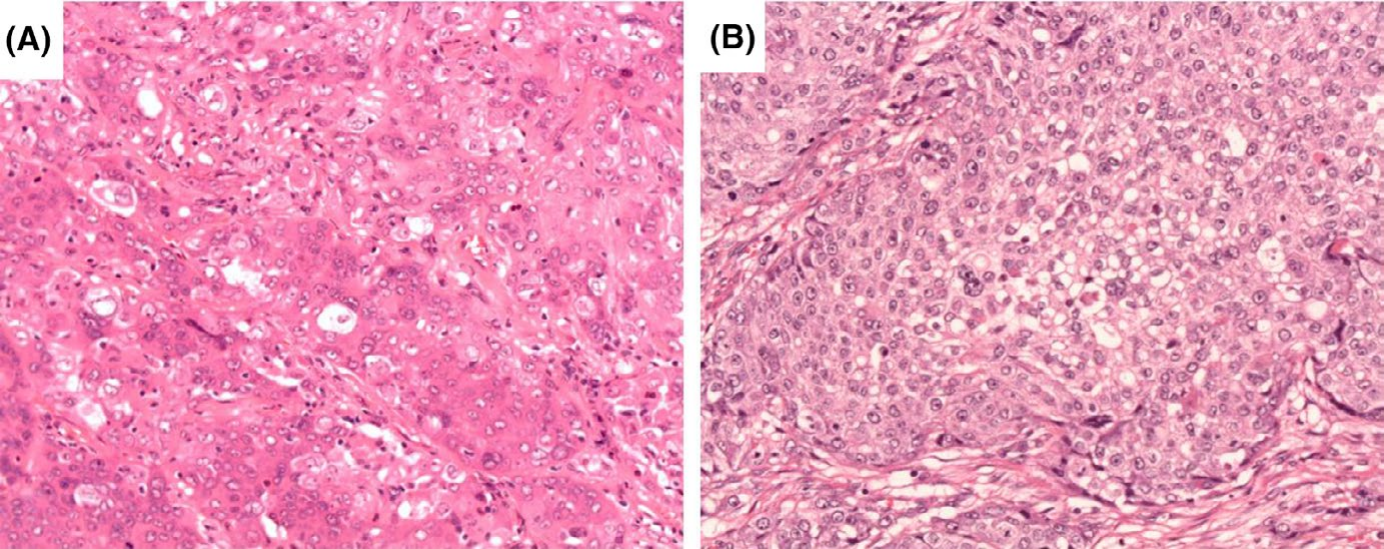
Pathological finding. A, Transverse colon cancer (histology (H&E ×100). Atypical epithelium was proliferating and forming nest‐like structure, analogous to the lung cancer histology (H&E ×100). B, The lung cancer histology resected 1 y ago (H&E ×100)

The resected specimen was a well‐circumscribed tumor measuring 6.5 × 5.5 cm in the transverse colon (Figure 3A and B), and histopathological finding was carcinoma without any specific differentiation (Figure 4A). The tumor was exposed onto the serosa, and intense lymphatic invasion was observed. Immunohistochemistry was positive for keratin AE1/AE3 and CK7, and negative for CK20, p40, and CDX2, resembling the tissue of the previously resected lung cancer (Figure 4B). The final diagnosis of lung cancer metastatic to the colon was confirmed.

Postoperative course was uneventful, and no gastrointestinal bleeding or progression of anemia was observed, even after he was restarted on antithrombotics. The patient was discharged on the thirteenth postoperative day. He died from systemic deterioration on the 40‐second postoperative day.

## 3 DISCUSSION

Mortality related to lung cancer is high, and it is the primary cause of cancer death worldwide.^1^ Of all cancer types, mortality for lung cancer is first among males and second among females worldwide.^1^ The common metastatic sites originating from lung cancer include lung itself, liver, brain, adrenal gland, and bone; however, metastasis to colon and rectum is quite rare.^2^ Kim et al reported that of 5239 patients with lung cancer, only 10 (0.19%) had metastasis to colon and rectum,^3^ and Lee et al showed that 21 of 8159 patients with lung cancer (0.26%) were diagnosed with gastrointestinal metastasis.^4^ Among those, 11 patients (52%) developed hemorrhage, and it was the leading cause of clinical presentation.^4^ In our case, the patient showed no apparent hematochezia, however, considering positive fecal occult blood test and anemia, the anemia can be attributed to the hemorrhage from the colonic metastatic lesion. Therefore, fecal occult blood test may be useful to find colonic metastasis for its low invasiveness and low cost.

Surgical intervention to manage gastrointestinal metastasis from lung cancer is becoming more and more important since the prognosis for lung cancer patients is becoming better with longer survival than before with the advent of precision medicine, such as tyrosine kinase inhibitors for lung adenocarcinoma with EGFR mutations.^5^

To care for patients with advanced stage lung cancer, early and systematic integration of palliative care is important to improve quality of life, as shown in a randomized controlled trial.^6^ Interestingly, Temel et al reported that early intervention was not only significantly useful for patient's health but also improved the duration of survival.^7^ Although that study aimed to control the patient's status nonsurgically, when gastrointestinal metastasis occured, surgical intervention was sometimes required.

Surgical intervention can be separated into palliative intent and curative intent. If the metastasis from lung cancer is confined to one organ, such as small bowel 8 or brain,^9^ surgery can be curative and survival benefits may be expected. On the other hand, if the systemic dissemination exists, as is frequently seen, surgical intervention is palliative. Clearly, metastasis must be detected early, and surgical intervention may be necessary to maintain quality of life.

Generally, during follow‐up for patients with lung cancer, CT to detect metachronous metastasis is only focused from the thoracic cavity to the upper abdomen, so lesions in the lower abdomen tend to be missed. When a patient had anemia or any abdominal symptoms, an aggressive evaluation to detect any lesion in the gastrointestinal area is strongly recommended. The colonoscopy is the golden standard for the diagnosis of colonic lesion. Upon diagnosis, cases with metachronous lung cancer metastatic to colon need to be differentiated from primary colorectal cancer.

There have been no reports regarding morphological characteristics of metastasis to colon.^10^ Theoretically, lung cancer mainly causes metastasis by hematogenous or lymphatic spread, so the cancer cells originally colonize to the submucosal or muscularis propria layer with rich vessels and proliferate. Therefore, macroscopic morphology tends to present a submucosal tumor at first, but as it protrudes toward the intestinal lumen, it becomes indistinguishable from primary colorectal cancer.

Immunohistochemistry is usually performed to discriminate between cancers from various origins. Among those, the expression pattern of CK7, CK20, CDX2, and TTF‐1 has been proven to be diagnostic.^10^ Primary colorectal cancer generally shows positive for CDX2 and CK20, and negative for CK7. Lung cancer is usually positive for TTF‐1 (squamous cell carcinoma is usually negative as in our case), and CK7, and negative for CK20. Our case was positive for CK7, and negative for CK20, CDX2, and TTF‐1. Therefore, preoperative diagnosis was lung cancer metastatic to colon rather than primary colon cancer.

In conclusion, prognosis after surgery for colonic metastasis from lung cancer is generally poor with only very slight chance of survival, even if with a curative intent. The surgical indication must be made by deliberate informed consent with the patient. Finally, surgeons are expected to be involved in the palliative care team, ideally making them available for timely surgical consultation and early diagnosis.

## CONFLICT OF INTEREST

I declare that I have no competing interests.

## AUTHOR'S CONTRIBUTIONS

RK and TS: served as the primary investigator and contributed to conceptualization and data collection. RK: drafted the manuscript. All authors have read and approved this manuscript for publication.

## DATA AVAILABILITY

The data supporting the conclusion are included in this article.

## ETHICS APPROVAL

Not applicable.

## CONSENT FOR PUBLICATION

Written informed consent was obtained from the patient for publication of this case report and any accompanying images.
